# Supplementary material for: Assessment of neuropsychological function in brain tumor treatment: a comparison of traditional neuropsychological assessment with app-based cognitive screening
Source: Acta Neurochir (Wien). 2022 Mar 1;164(8):2021–34. doi: 10.1007/s00701-022-05162-5 (PMC9338148; doi:10.1007/s00701-022-05162-5)
Supplement: Supplementary file 1 — Supplementary file1 (DOCX 5210 KB) [file 701_2022_5162_MOESM1_ESM.docx]

**Assessment of neuropsychological function in brain tumour treatment: A comparison of traditional neuropsychological assessment with app-based cognitive screening**

**Supplementary material**

Rafael Romero-Garcia^1*^, Mallory Owen^1*^, Alexa McDonald^2^, Emma Woodberry^2^, Moataz Assem^3^, Pedro Coelho^4^, Rob C Morris^5^, Stephen J Price^5^, Tom Santarius^5,6^, John Suckling^1,7,8^, Tom Manly^8^, Yaara Erez^3^, Michael G Hart^1^

*****These authors contributed equally

1. University of Cambridge School of Clinical Medicine, 2. Department of Neuropsychology, Cambridge University Hospitals NHS Foundation Trust, 3. MRC Cognition and Brain Sciences Unit, University of Cambridge, 4. Neurophys Limited, 5. Department of Neurosurgery, Addenbrooke’s hospital, Cambridge, 6. Physiology, Development and Neuroscience, University of Cambridge, 7. Behavioural and Clinical Neuroscience Institute, University of Cambridge; 8. Cambridge and Peterborough NHS Foundation Trust.

**Figure S1.** Distribution of z-scores for individual items across patients grouped by major domains (i.e., each count represents the score of one item in one of the patients’ assessment) Values less than 0 indicate items where brain tumour patients showed lower performance than healthy controls.

**
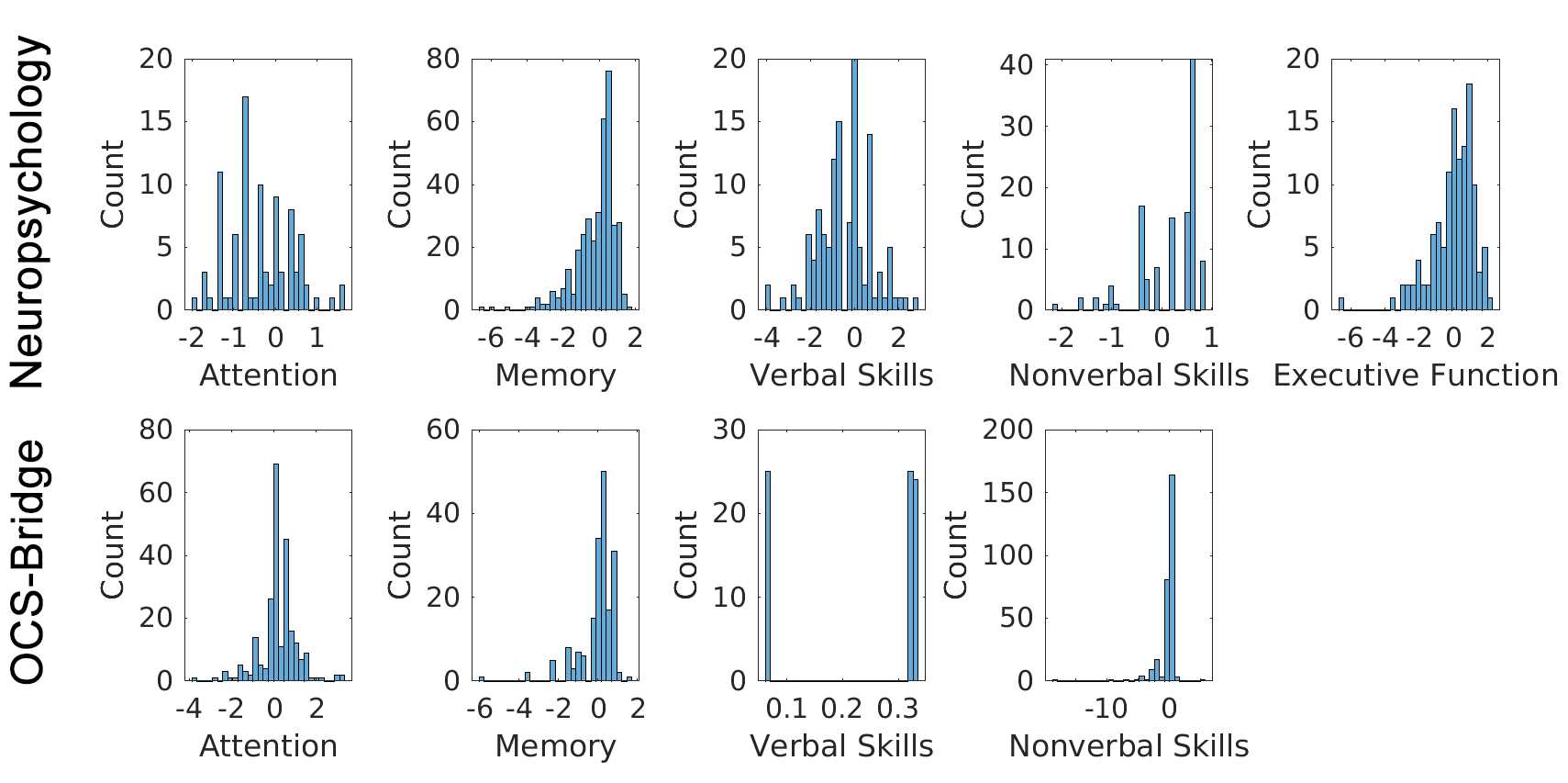
**

**Table S1**. Individual cognitive tests included in the OCS-Bridge assessment and the traditional neuropsychology battery

| **OCS-Bridge Assessment** | |  | **Neuropsychology Battery** | |
| --- | --- | --- | --- | --- |
| **Oxford Cognitive Assessment** | |  | **Domain** | **Test** |
| **Domain** | **Test** |  | Verbal Memory | AMIPB Story – Immediate Recall |
| Attention | Hearts Accuracy |  | Verbal Memory | AMIPB Story – Delayed Recall |
| Attention | Space Neglect |  | Verbal Memory | BMIPB Word List - A1-A5 |
| Attention | Space Neglect |  | Verbal Memory | BMIPB Word List - A6 |
| Attention | Object Neglect |  | Verbal Memory | BMIPB Word List - Word recognition |
| Attention | Object Neglect |  | Verbal Memory | BMIPB Word List - List recognition |
| Attention | Perseveration |  | Nonverbal Memory | BMIPB Complex Figure – Immediate Recall |
| Attention | Hearts Organization Index |  | Nonverbal Memory | BMIPB Complex Figure – Delayed Recall |
| Perception | Visual PerceptionTotal |  | Nonverbal Memory | BMIPB Design Learning - A1-A5 |
| Perception | Visual Field |  | Nonverbal Memory | BMIPB Design Learning - A6 |
| Perception | Visual Field |  | Nonverbal Memory | BMIPB Design Learning - Design Recognition |
| Perception | Visual Extinction Left |  | Nonverbal Memory | BMIPB Design Learning - Design Identification |
| Perception | Visual Extinction Right |  | Verbal Skills | Test of Premorbid Functioning |
| Memory | Memory Free Verbal |  | Verbal Skills | Letter Fluency – FAS |
| Memory | Overall Verbal Memory |  | Verbal Skills | Semantic Fluency – Animals |
| Memory | Episodic Memory |  | Verbal Skills | Graded Naming Test |
| Memory | Orientation |  | Verbal Skills | Syntactic Speech Comprehension |
| Language | Picture Naming |  | Nonverbal skills | BMIPB Complex Figure copy |
| Language | Semantics |  | Nonverbal skills | VOSP Object Decision |
| Language | Sentence Reading Words Recalled |  | Nonverbal skills | VOSP Number Location |
| Praxis | Hand Positions |  | Nonverbal skills | VOSP Cube Analysis |
| Praxis | Finger Positions |  | Attention | WAIS-IV Digit Span - Forward |
| Praxis | Overall |  | Attention | WAIS-IV Digit Span - Backward |
| Number | Calculation |  | Executive Function | Initiation - Time |
| Number | Number Writing |  | Executive Function | Inhibition - Time |
|  |  |  | Executive Function | Inhibition - Score |
| **Cambridge Assessment** |  |  | Executive Function | Brixton |
| Perception | Boat Visual Acuity |  | Mood | Beck Anxiety Inventory |
| Perception | Line bisection |  | Mood | Beck Depression Inventory |
| Perception | Line bisection |  |  |  |
| Perception | Postcards object perception |  |  |  |
| Attention | SALT sustained attention |  |  |  |
| Attention | Target detection |  |  |  |
| Attention | Consistency of Attention |  |  |  |
| Attention | Target space bias |  |  |  |
| Attention | Target space bias |  |  |  |
| Attention | Reaction time space bias |  |  |  |
| Attention | Reaction time space bias |  |  |  |
| Verbal Working Memory | Forward Digit Span |  |  |  |
| Verbal Working Memory | Backwards Digit Span |  |  |  |
| Prospective Memory | Prospective Memory |  |  |  |
| Prospective Memory | Retrospective Memory |  |  |  |

**Table S2.** Combined domains across neuropsychological testing and OCS-Bridge testing.

| **Combined Domain** | **Neuropsychology** | **OCS-Bridge** |
| --- | --- | --- |
| Attention | Attention | Attention |
| Memory | Nonverbal Memory | Memory |
|  | Verbal Memory | Prospective Memory |
|  |  | Verbal Working Memory |
| Verbal Skills | Verbal Skills | Language |
| Nonverbal Skills | Nonverbal Skills | Praxis |
|  |  | Number |
|  |  | Perception |
| Executive Function | Executive Function | - |
| Mood | Mood | - |

**Table S3.** OCS-Bridge screening and neuropsychological assessments completed by each participant.

**Table S4.** Average total number of cognitive deficits and standard deviations across patients for each domain and assessment. A positive T value indicates a larger number of deficits detected by traditional neuropsychology compared with OCS-BRIDGE.

|  | | Neuropsychology  Average (SD) | OCS-BRIDGE  Average (SD) | *T* value | *P* value |
| --- | --- | --- | --- | --- | --- |
| Attention | Preoperative | 0.765 (0.83) | 0.353 (0.79) | 1.48 | 0.15 |
|  | Postoperative | 0.786 (0.70) | 0.500 (1.07) | 0.76 | 0.46 |
| Memory | Preoperative | 0.941 (1.20) | 0 (0) | 3.24 | **0.003** |
|  | Postoperative | 1.571 (1.70) | 0.125 (0.35) | 2.36 | **0.03** |
| Non-verbal skills | Preoperative | 0 (0) | 0.471 (0.62) | -3.11 | **0.004** |
|  | Postoperative | 0 (0) | 1.125 (1.36) | -3.16 | **0.005** |
